# Supplementary material for: Bacterially synthesized tellurium nanostructures for broadband ultrafast nonlinear optical applications
Source: Nat Commun. 2019 Sep 4;10:3985. doi: 10.1038/s41467-019-11898-z (PMC6726626; doi:10.1038/s41467-019-11898-z)
Supplement: Supplementary file 1 — Supplementary Information [file 41467_2019_11898_MOESM1_ESM.pdf]

Supplementary Information – Wang et al.

Bacterially Synthesized Tellurium Nanostructures for Broadband Ultrafast Nonlinear Optical Applications

Kangpeng Wang,[1,2] Xiaoyan Zhang, [1] Ivan M. Kislyakov,[1] Ningning Dong, [1] Saifeng Zhang, [1] Gaozhong Wang,[1,3] Jintai Fan, [1] Xiao Zou,[4] Juan Du,[4] Yuxin Leng, [4] Quanzhong Zhao,[4] Kan Wu, [5] Jianping Chen, [5] Shaun M. Baesman,[6] Kang-Shyang Liao,[7] Surendra Maharjan,[7] Hongzhou Zhang, [3] Long Zhang, [1,4,8]* Seamus A. Curran,[7] Ronald S. Oremland,[6] Werner J. Blau,[3] and Jun Wang,[1,4,8]**

1. Laboratory of Micro-Nano Optoelectronic Materials and Devices, Laboratory of Laser and Infrared Materials, Key Laboratory of Materials for High-Power Laser, Shanghai Institute of Optics and Fine Mechanics, Chinese Academy of Sciences, Shanghai 201800, China.
2. Department of Electrical Engineering, Technion-Israel Institute of Technology, Haifa 3200003, Israel
3. School of Physics, CRANN and AMBER Research Centres, Trinity College Dublin, Dublin 2, Ireland
4. State Key Laboratory of High Field Laser Physics, CAS Center for Excellence in Ultra-intense Laser Science, Shanghai Institute of Optics and Fine Mechanics, Chinese Academy of Sciences, Shanghai 201800, China
5. State Key Laboratory of Advanced Optical Communication Systems and Networks, Department of Electronic Engineering, Shanghai Jiao Tong University, Shanghai 200240, China
6. US Geological Survey, Menlo Park, CA 94025, USA
7. Institute for NanoEnergy, Department of Physics, University of Houston, Houston, TX 77204, USA.
8. Center of Materials Science and Optoelectronics Engineering, University of Chinese Academy of Sciences, Beijing 100049, China

*Corresponding Email:

jwang@siom.ac.cn (J.W.)

kanwu@sjtu.edu.cn (K.W.)

lzhang@siom.ac.cn (L.Z.)

Supplementary Note 1: Fabrications of Bio-Te composite and samples for comparison

To fabricate Bio-Te-PmPV composite, PmPV powder was firstly dissolved in toluene to a concentration of 0.5 g/L. Subsequently, the Te micro-pellets were added into the polymer solutions, followed by gentle stirring for 24 h. The concentration of Te was adjusted from 0.03 to 0.20 wt.%. Similarly, chemically synthesized Te (Chem-Te) (Sigma-Aldrich) was dispersed by PmPV in toluene to form Chem-Te-PmPV composite as the reference sample.

The PmPV had a bright yellow color when dissolved in toluene. To avoid the influence of PmPV while achieving the best Te dispersion, the concentration of PmPV used to disperse Te was controlled at a very low level (0.5 g/L in toluene). After blending with Te (0.05 wt.%), the color of the mixture turned from bright yellow to green-gray and became deeper with the increase of stirring time. This phenomenon implied strong interactions between PmPV chains and BioTe nanocrystal, as well as an effective exfoliation of the Bio-Te aggregates. For comparison, a poor dispersion was observed in the Te-toluene mixture that lacked PmPV.

To prepare the graphene-NMP dispersion for comparison experiments, the graphite powder was immersed in NMP at a concentration of 0.1 mg/mL and sonicated for 2h. Then followed by centrifugation at 1500 rpm for 30 min and the bottom 1/4 dispersion were removed. The resultant dispersions were diluted by NMP to adjust its linear transmission for further optical experiments.

For the graphene-PMMA films, the graphene-PMMA toluene (4mt% PMMA) dispersions were produced by firstly stirring for 2h using ultrasonic bath then magnetically stirring for 48 h with an initial concentration of graphene as 5 mg/ml. The resultant dispersions were centrifuged at 1500 rpm for 30 min to remove unexfoliated or aggregated powders. The top 3/4 centrifuged dispersions were gently extracted by pipetting. The obtained graphene-PMMA films were manufactured by solution-cast method by transferring the homogeneous solutions into glass petri-dishes with a diameter ~60 mm. Then after drying at 50°C for 2 days, we obtained the high-quality transparent films with uniform surfaces. The WS_2-PMMA films were fabricated in the same procedures. For ellipsometer measurements, the procedures were same except drying on silicon wafer instead of petri-dishes.

Supplementary Note 2: Nonlinear scattering in Bio-Te suspension and carbon materials

There are two processes in in a nanoparticle suspension that can determine the strength of nonlinear scattering: (1) evaporation of the liquid and (2) phase transitions of nanoparticles by laser-induced heat. The evaporation of liquid occurs because the nanoparticles absorbs laser energy and evaporate the surrounding solvent to micro-bubbles. The local temperature increase of a nanoparticle, ΔT, is proportional the ratio of its absorption cross-section σ to the heat capacity C_p, that is, $\Delta T \propto \sigma/C_p$ [1, 2]. The σ/C_p ratio of Te to that of graphite, $(\sigma/C_p)_{Te}$ / $(\sigma/C_p)_{graphite}$, is

estimated to be ~4.87 (see below about the calculation procedures). This indicates that Te nanoparticles may heat up ~4.9 times more than graphite at the same applied irradiance. The phase transitions of nanoparticles can also generate microbubbles by themselves. For Te nanocrystals, there are two phase-transitions: melting at 450°C, and boiling at 988°C. For carbon materials, however, only sublimation is exhibited at a much higher temperatures (up to ~4000°C at ambient pressure)[3]. Therefore, for optical limiting, the Te nanocrystals can contribute a much larger amount of micro-inhomogeneities (droplets and bubbles) than carbon materials because of the two Te phase transitions.

The σ/C_p for Te and Carbon Materials can be calculated as follows: The linear absorptive coefficient α_0 has the relationship $\alpha_0 = N\sigma \cong \rho\sigma N_A/M$, where the N is the absorber particle density, σ is the absorptive cross-section, ρ is the material density, N_A is the Avogadro constant and M is the molar mass. From the literature[3], we know that the absorption coefficient α_0 of graphite is ~2.21×10^3 cm^{-1} and that for tellurium is ~8.43×10^3 cm^{-1} at 1 eV photon energy. The molar thermal capacity, C_p, of Te and graphite is respectively 25.73 J/mol/K and 8.517 J/mol/K; the molar mass, M, of Te and graphite is 127 g/mol and 12 g/mol; and the density ρ of Te and graphite is 6.3 g/cm^3 and 2.3g/cm^3. For thsi data, the $(\sigma/C_p)_{Te}$ / $(\sigma/C_p)_{graphite}$ ratio is calculated to be ~4.87.

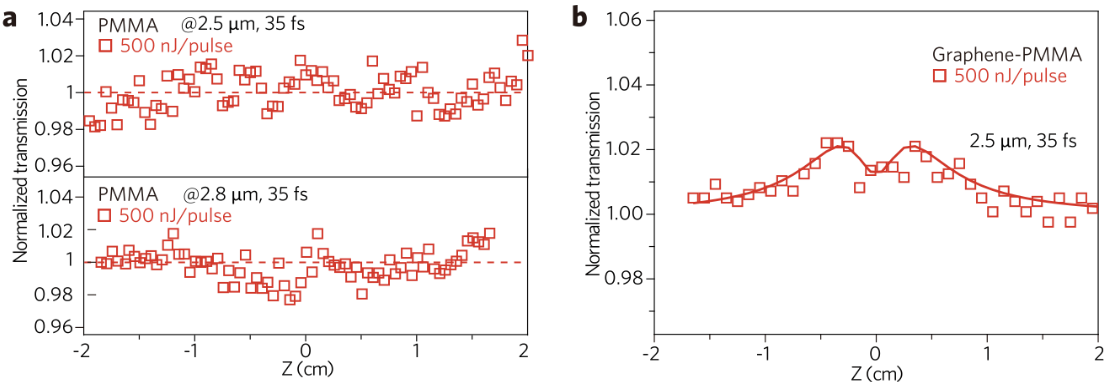

Supplementary Figure 1 | Mid-infrared open-aperture performance of PMMA samples. (a) Pure PMMA films, showing no detectable NLO response of PMMA at 2.5 μm and 2.8 μm wavelengths. (b) Graphene-PMMA films at 500 nJ/pulse high irradiance, exhibiting both saturable absorption at z $\pm$~ 4 mm and two-photon absorption at around $z \cong 0$ mm.

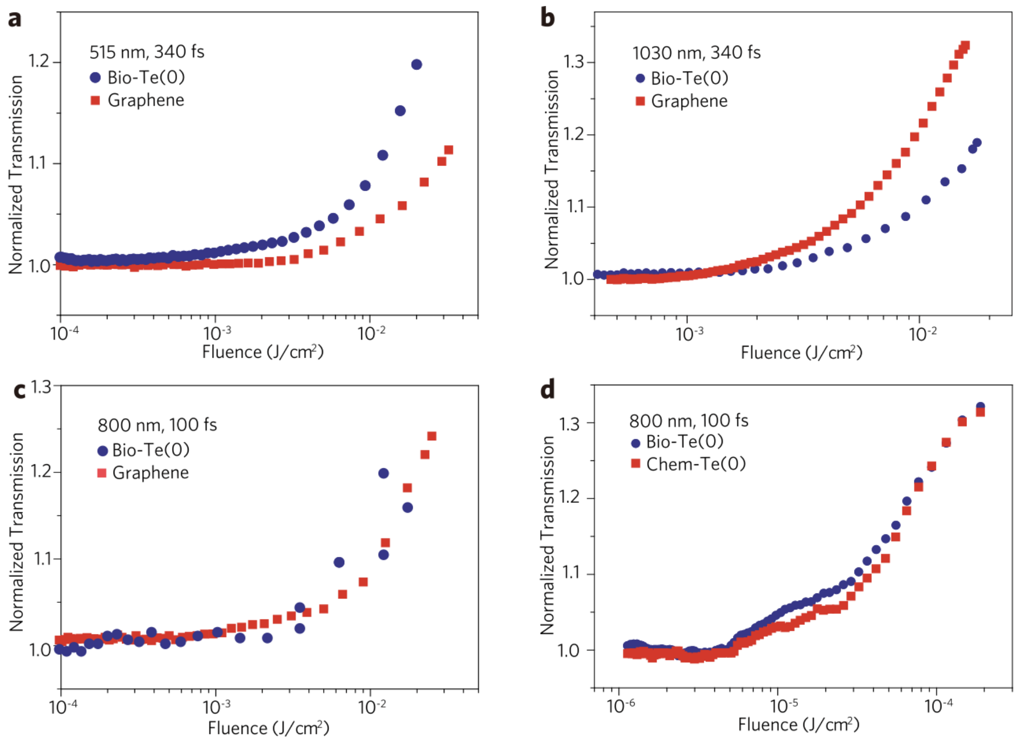

Supplementary Figure 2 | Comparisons of the NLO performances: Bio-Te-PmPV, Chem-Te-PmPV, and graphene dispersions in NMP. (a, b) for 340 fs pulses at 515 nm and 1030 nm and (c, d) for 100 fs pulses at 800 nm, respectively.

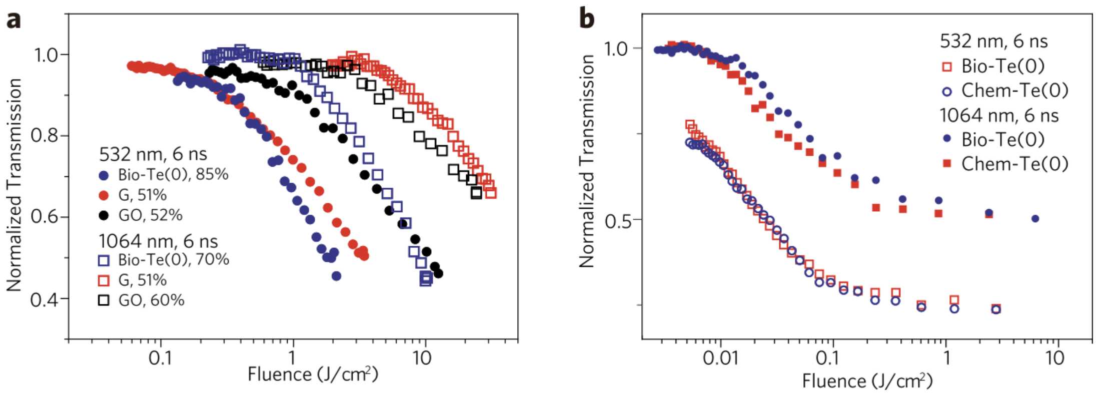

Supplementary Figure 3 | Comparison of NLO performance for Te-PmPV composites to several typical NLE materials. (a) Bio-Te-PmPV, graphene and graphene oxide (GO) dispersions under 532 and 1064 nm 6 ns irradiations; (b) Bio-Te- and Chem-Te-PmPV composites.

Supplementary Note 3: Details of Bio-Te all-optical switch

The Bio-Te thermo-optical switch is based on the polarization interference. When the Bio-Te nanocrystal absorbed the energy of the control beam, a change in its refractive index was introduced owing to the thermo-optical effect. Because Bio-Te film was anisotropic in thickness, the phase shift caused by the change of the refractive index was also directional, that is, the phase shift was polarization-dependent. We named the two-orthogonal axes as x and y, respectively, which corresponded to the polarizations for which the incident light had the maximum and minimum phase shift, respectively. As a result, the two components of the signal beam along the x and y polarization directions experienced different phase changes after interacting with the sample, and their phase difference was determined by the control beam. Finally, the two components interfered with each other when they reached the polarizer and the signal output after the polarizer was controlled by the availability of the pump pulsed light.

The thermo-optic coefficient of Bio-Te nanocrystals can be estimated as follows: the contribution is approximately proportional to the ratio of the optical intensity distribution, and in a thin film, this is the ratio of the material volume [4]. In our Bio-Te-PMMA film, the volume ratio of Bio-Te-PMMA is ~1:100. Then the contribution to the refractive index change can be approximately given by $\Delta n \cong 1\% \cdot \Delta n_{\mathrm{Te}} + 99\% \cdot \Delta n_{\mathrm{PMMA}}$. Knowing that the thermo-optic coefficient for pure PMMA[5] is -1.3×10^{-4} K^{-1} and is -1.64×10^{-4} K^{-1} for Bio-Te-PMMA film, the thermo-optic coefficient of Bio-Te can be estimated to be approximately -3.5×10^{-3} K^{-1}.

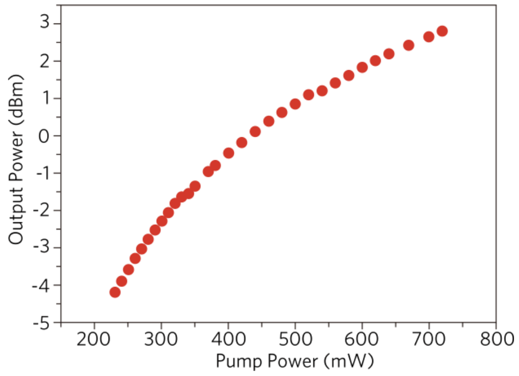

Supplementary Figure 4 | Output power in Bio-Te mode-locked EDF laser. The lasing action was observed from 225 mW pump power, and the maximum mode-locked output power was measured to be 2.95 dBm (1.95 mw) with 720 mW pump power.

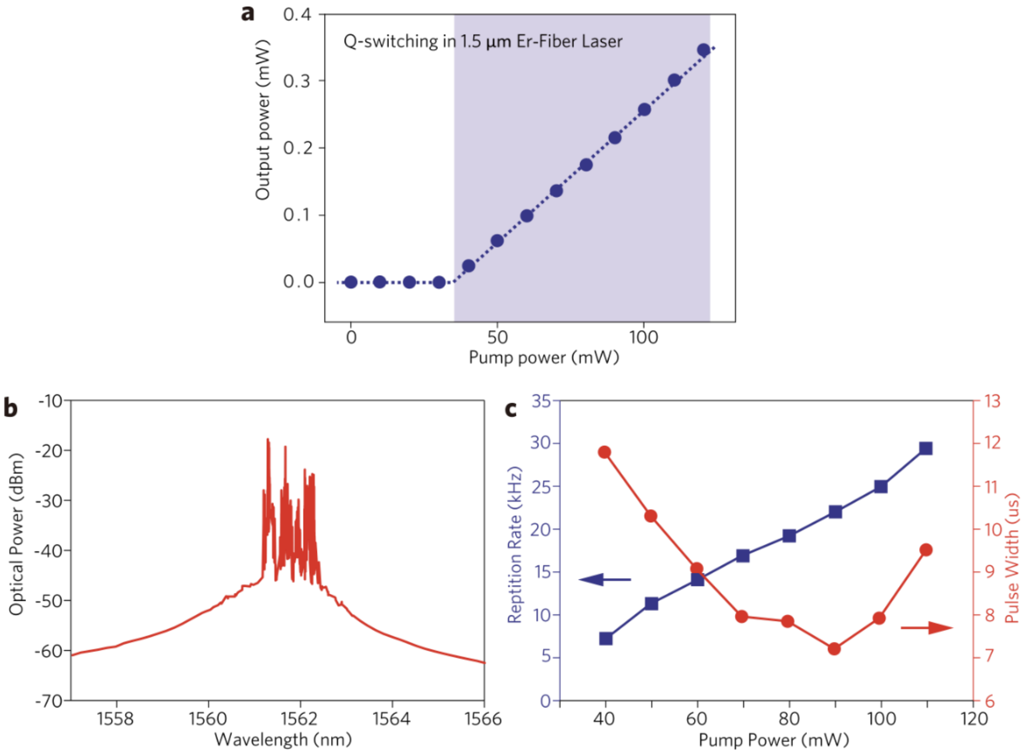

Supplementary Figure 5 | Q-Switching operation in an EDF laser: (a) average pulse power as a function of pump power; (b) the corresponding pulse spectrum; and (c) the repetition rate (left) and pulse duration (right) of Q-switching pulses as a function of the 980 nm pump power.

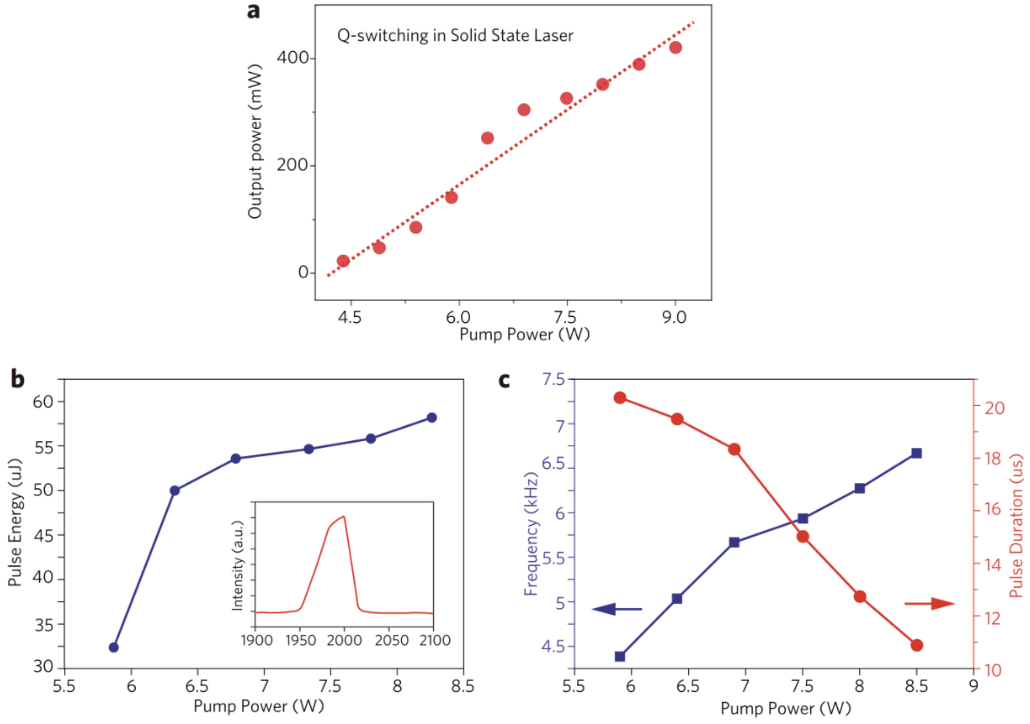

Supplementary Figure 6 | Q-Switching operation in Tm:YAP 2 µm laser: (a) average pulse power as a function of pump power; (b) average pulse power as a function of pump power. (inset: corresponding pulse spectrum); and (c) evolution of pump power and the repetition rate (left) or pulse duration (right) of a Q-switching pulse.

Supplementary Table 1 | Photoluminescence lifetimes of Bio-Te-PmPV and PmPV.

**Wavelength (nm)**	**Samples**	**τ_1 (ps) (proportion)**	**τ_2 (ps) (proportion)**	**τ_3 (ns) (proportion)**
528	PmPV	460 (35.52 %)	1.11 (53.69 %)	4.53 (10.80 %)
	Bio-Te-PmPV	338 (36.13 %)	1.09 (49.33 %)	4.99 (14.54 %)

Fitted parameters for the photoluminescence kinetics under the excitation at 528 nm using exponential decay model, showing the influence of Bio-Te nanoparticles to photoluminescence lifetimes.

Supplementary References

1. Kislyakov, I. M., Nunzi, J.-M., Zhang, X., Xie, Y., Bocharov, V. N. & Wang, J., Stimulated Brillouin scattering in dispersed graphene. *Optics Express* **26**, 34346-34365 (2018).

2. McEwan, K. & Madden, P. A., Transient grating effects in absorbing colloidal suspensions. *The Journal of Chemical Physics* **97**, 8748-8759 (1992).

3. Haynes, W. M., *CRC Handbook of Chemistry and Physics*. (CRC Press, New York, 2016).

4. Wu, K., Wang, Y., Qiu, C. & Chen, J., Thermo-optic all-optical devices based on two-dimensional materials. *Photonic Research* **6**, C22-C28 (2018).

5. Zhang, Z., Zhao, P. & Lin, P., Thermo-optic coefficients of polymers for optical waveguide applications. *Polymer* **2006,** 47, (14), 4893-4896.
